# Dissecting the Multiplicity of Immune Effects of Immunosuppressive Drugs to Better Predict the Risk of *de novo* Malignancies in Solid Organ Transplant Patients

**DOI:** 10.3389/fonc.2019.00160

**Published:** 2019-03-27

**Authors:** Michela Cangemi, Barbara Montico, Damiana A. Faè, Agostino Steffan, Riccardo Dolcetti

**Affiliations:** ^1^Immunopathology and Cancer Biomarkers, Translational Unit, Centro di Riferimento Oncologico di Aviano (CRO), IRCCS, Aviano, Italy; ^2^Translational Research Institute, University of Queensland Diamantina Institute, Brisbane, QLD, Australia

**Keywords:** immunosuppressive drugs, solid organ transplant, dendritic cells, cancer, immune cells

## Abstract

*De novo* malignancies constitute an emerging cause of morbidity after solid organ transplant (SOT), significantly affecting the long-term survival of transplant recipients. Pharmacologic immunosuppression may functionally impair the immunosurveillance in these patients, thereby increasing the risk of cancer development. Nevertheless, the multiplicity and heterogeneity of the immune effects induced by immunosuppressive drugs limit the current possibilities to reliably predict the risk of *de novo* malignancy in SOT patients. Therefore, there is the pressing need to better characterize the immune dysfunctions induced by the different immunosuppressive regimens administered to prevent allograft rejection to tailor more precisely the therapeutic schedule and decrease the risk of *de novo* malignancies. We herein highlight the impact exerted by different classes of immunosuppressants on the most relevant immune cells, with a particular focus on the effects on dendritic cells (DCs), the main regulators of the balance between immunosurveillance and tolerance.

## Introduction

Solid organ transplant (SOT) is an established procedure for patients with end-stage disease and the availability of potent anti-rejection drugs (1) significantly reduced the occurrence of acute and chronic allograft rejections, even though long-term survival is still unsatisfactory. Indeed, viral infections/reactivations, cardiovascular complications and tumor onset are among the major causes of morbidity and mortality in SOT patients (2, 3). In particular, SOT recipients have a 2 to 5-fold higher risk to develop a *de novo* neoplasm than the general population (4, 5). The tumor types with the highest risk relative to the general population are Kaposi sarcoma, lip carcinoma, non-melanoma skin cancers, non-Hodgkin lymphoma, liver, vulvar, and anal carcinoma (4, 5). Notably, the majority of these cancers are pathogenically related to oncogenic viruses, including Human Herpesvirus 8 (HHV8), Epstein-Barr Virus (EBV), Human Papillomaviruses (HPV), and Hepatitis B and C (3), whose control by host immune system is impaired in the transplant setting. Skin cancers are the most frequent malignancy observed in SOT recipients, being observed in 8% of patients. The high incidence of skin cancers has been related to the high mutation burden due to UV exposure. These tumors, which have enhanced immunogenicity due to UV-induced mutations, are poorly controlled in immunosuppressed SOT recipients, thus explaining their increased incidence in this setting as compared to the general population. Other virus-unrelated malignancies such as carcinomas of the breast and prostate are not increased in transplant recipients. Post-transplant malignancies are often characterized by high aggressive clinical features and poor prognosis, thus representing an important medical need (6). Although iatrogenic immunosuppression has the power to inhibit the rejection of the transplanted organ, this treatment may limit the ability of patients' immune system to control nascent and overt tumors. Immune-evasion plays a pivotal role in tumorigenesis in the transplant setting, being directly promoted by the immunosuppressive effects of the drugs used and indirectly favored by the increased rate of oncogenic virus infections and reactivations, which may further contribute to impair host immune functions. The main mechanisms that drive the onset of *de novo* tumors in SOTs can be grouped into three major categories: (1) direct pro-oncogenic properties of select immunosuppressive drugs; (2) increased risk of oncogenic virus reactivation; (3) impaired immunosurveillance of tumor cells (7).

The most frequent *de novo* tumors arising after transplantation include Non-melanoma skin cancers (NMSC) (8, 9), often associated with Human papilloma virus (HPV) infection (10), Merkel cell carcinomas (MCC) (11, 12), related to Merkel cell polyomavirus (MCV) (13), post-transplant lymphoproliferative disease (PTLD), associated with Epstein-Barr Virus (EBV) (14), and Kaposi's sarcoma (KS), driven by Human Herpesvirus-8/KS herpesvirus infection (15).

If on one side SOT is the only treatment available for some end-stage diseases, on the other hand, the duration and type of immunosuppression can increase the risk of *de novo* malignancies in these patients. This may be at least in part due to the defective immune control of infections and/or reactivation by oncogenic viruses. Nevertheless, emerging evidence indicates that the various immunosuppressive drugs and regimes administered to SOT patients may have heterogeneous and still poorly defined effects on immune cell populations that may variably affect the cancer immunosurveillance (16) in these patients. On these grounds, the immune effects of immunosuppressive drugs may ultimately dictate the extent of risk to develop a *de novo* malignancy in SOT recipients.

On these grounds, there is the pressing need to better characterize the immune dysfunctions related to the immunosuppressive treatment of these patients to better understand the impact of the various immunosuppressive drugs on the immune system and how the chronic use of these drugs may favor the tumor onset in SOT patients. This may ultimately lead to a more precise and safe tailoring of the immunosuppressive schedule and limit as much as possible the risk of cancer development in these patients. The purpose of this review is to highlight the impact exerted by different classes of immunosuppressants on the immune system, with a particular focus on the effects on dendritic cells (DCs) and their central role in orchestrating both tolerance and anti-tumor immunity.

## Immunosuppressive Drugs in Solid Organ Transplantation and Their Relative Risk of Cancer Development

### Corticosteroids

Corticosteroids are a class of steroid hormones used primarily to reduce inflammatory and immune responses in various clinical conditions, and constitute an important component of the immunosuppressive regimens administered to SOT recipients. These drugs exert their effects by binding to an intracellular receptor, which then act to modulate gene transcription in target tissues, also including genes regulating immune responses. After binding to glucocorticoid receptors (GR) in the cytoplasm, corticosteroids inhibit the nuclear translocation and function of transcription factors, such as Activator Protein 1 (AP1) and Nuclear Factor-κB (NF-κB), resulting in a decreased inflammatory response through inhibition of pro-inflammatory cytokines such as interleukin (IL)-1, IL-2, IL-6, interferon (IFN)-γ and tumor necrosis factor (TNF)-α (17, 18). These drugs may also induce the production of anti-inflammatory proteins, including lipocortin and the inhibitor of NF-κB (IκB). Evidence accumulated so far clearly indicates that most of the immune effects triggered by corticosteroids are due to their ability to induce apoptosis of immune cells, particularly T lymphocytes and monocytes/macrophages. The possible contribution of corticosteroids to the risk of cancer development in transplanted patients is still unclear. It has been suggested that the ability of these drugs to promote anti-apoptotic and proliferative effects in various cell types (19, 20) could increase the risk of a *de novo* malignancy in SOT patients, although the specific contribution of corticosteroids is difficult to assess considering that these drugs are often administered in combination with other immunosuppressive agents.

A systematic review of the effects related to steroid avoidance and withdrawal after kidney transplantation did not disclose significant differences in the occurrence of malignancies in these patients up to 5 year after transplantation (21). Nevertheless, long-term consequences of steroid avoidance and withdrawal remain still unclear and prospective long-term studies are needed to draw definite conclusions.

### Antimetabolites

#### Azathioprine

Azathioprine (AZA) was the first anti-proliferative agent available for clinical use and is commonly used in SOT patients in combination with other drugs, mainly Cyclosporine and Prednisone. AZA is converted to 6-mercaptopurine (6-MP) *in vivo*, which in turn is converted into 6-thiouric acid, 6-methyl-MP, and 6-thioguanine. These metabolites are incorporated into the DNA, block the *de novo* pathway of purine synthesis and inhibit cell proliferation. The main toxic effects induced by AZA are leukopenia, thrombocytopenia, anemia, and hepatotoxicity (22). Immunosuppressive function of AZA is due to its ability to negatively interfere with the function and proliferation of T and B lymphocytes, with a relatively higher selectivity for T cells. In addition, AZA was shown to photosensitize the skin (23), promoting the accumulation of 6-thioguanine in DNA, which results in enhanced production of mutagenic reactive oxygen species after exposure to ultraviolet A (UVA) (24). These effects were suggested to have an impact on the development of squamous cell carcinomas (SCC) and other skin cancers in transplanted patients. A meta-analysis showed that treatment with AZA was associated with a significantly increased risk of SCC in SOT patients, whereas no significant association was observed between exposure to AZA and basal cell carcinoma (BCC) (25). A more recent study reported a significantly elevated risk of both BCC and SCC in SOT patients treated with AZA (26). Notably, multivariate analysis disclosed that AZA was associated with significantly higher risk than mycophenolate mofetil, sirolimus, cyclosporine, or tacrolimus (26). A recent whole exome sequencing study (WES) on cutaneous SCC from immunosuppressed patients has revealed a high mutation rate with an average of 50 mutations per megabase pair DNA. In particular, mutational signature analysis reveals the presence of a novel signature, whose presence correlates with chronic exposure to AZA (27). This signature is probably the result of combined action of UV exposure and incorporation of AZA metabolites into DNA, ultimately promoting tumor progression. These findings have clinical relevance for patients under treatment with AZA, who should be counseled about their skin cancer risk and UV photosensitivity.

#### Mycophenolate Mofetil

Mycophenolic acid (MPA), isolated in 1896 from *Penicillium brevicompactum* by Bartolomeo Gosio, is the active metabolite of mycophenolate mofetil (MMF) (28). MMF is a potent immunosuppressive drug used especially in combination with Calcineurin inhibitors (CNIs). MPA is the reversible uncompetitive inhibitor of inosine monophosphate dehydrogenase (IMPDH), the enzyme that synthesizes *de novo* guanosine nucleotides, thereby inhibiting DNA synthesis. In 2000, MMF was approved by FDA for use in liver transplantation, and in 2005 it has been shown (29) that treatment with MMF improved graft and survival of adult liver transplanted patients. Common side effects are nausea, vomiting, diarrhea (30) and high risk of opportunistic infection, in particular due to Cytomegalovirus (CMV) (31, 32). As compared to the use of AZA, MPA was associated with significantly lower risk of cutaneous SCC in cohorts of kidney, kidney–pancreas, heart and lung transplant patients (33, 34), an effect probably related to a decreased ability of MMF to induce ultraviolet light-related transforming effects (35).

### Calcineurin Inhibitors

Calcineurin is a calcium/calmodulin-activated serine/threonine phosphatase that, once stimulated, de-phosphorylates and thereby activates members of the nuclear factor of activated T cells (NFAT) transcription factor family (36). Upon activation, NFAT family members migrate into the nucleus and activate transcription. Calcineurin is also responsible for the transcription of the genes encoding for IL-2 and several other cytokines, including TNF-α and IFN-γ. Calcineurin inhibitors (CNI), such as Cyclosporine A (CsA) and Tacrolimus (TAC), exert their immunosuppressive action through the inhibition of the Calcineurin pathway, inducing NFAT inhibition, which down-regulates IL-2 and INF-γ expression, and inhibits T-cell activation and proliferation in response to foreign antigens (37). Cyclosporine is a cyclic endecapeptide (38) commonly used to prevent rejection of liver, heart and kidney transplants. TAC, also called FK-506, is a macrolide antibiotic isolated from *Streptomyces tsukubaensi*. The use of this drug was initially restricted to patients with liver transplantation, but, more recently, it was also extended to patients with heart, pancreas and kidney transplantation (39, 40). Several studies reported increased risks of cancer related to the use of CNI (41, 42). Besides their ability to inhibit immune responses, CNI may also directly promote the aggressiveness and invasiveness of cancer cells by hampering antiviral immunity, supporting DNA-damage or up-regulating growth-promoting or pro-angiogenetic cytokines such as TGF-β, IL-10, or VEGF-2 (43–45). Indeed, adenocarcinoma cells treated with CsA *in vitro* were shown to undergo marked morphological and functional alterations, including increased cell motility and invasive growth (46). CNI also mediate activation of the Ras oncogene and promote renal cancer cell proliferation. Notably, VEGF overexpression induced by CSA in patients with renal carcinoma was dependent on Ras activation (47, 48). The pro-angiogenic effects of CNI-induced VEGF may probably constitute one of the major factors contributing to the increased rate of malignancies observed in transplanted patients treated with these drugs. Consistently, conversion from CNIs to Sirolimus (SRL) was shown to reduce the vascularization of cutaneous SCC in SOT patients (49). With regard to the risk of NMSC, CNIs were shown to cooperate with UVA and UVB in increasing the levels of TGF-β and suppressing p53 expression through the induction of ATF3 (50). Treatment with CSA was consistently associated with an increased incidence of EBV-driven post-transplant lymphoproliferative disorders (PTLDs), an effect probably dependent on the ability of CSA to induce EBV lytic replication in B lymphocytes and promote the release of the B-cell growth promoting cytokine IL-6 (51). Both CSA and TAC were shown to inhibit DNA repair (52), another possible contributory factor to the increased risk of cancer promoted by these drugs. The risk of *de novo* malignancies was increased in TAC-treated patients compared to that observed in patients treated with CsA (41).

### mTOR Inhibitors

Mammalian target of rapamycin (mTOR) inhibitors are a large class of drugs that inhibit mTOR, a serine-threonine kinase involved in cell growth, proliferation, protein synthesis, and apoptosis (53, 54). The PI3K/Akt/mTOR pathway is often up-regulated in various malignancies and mTOR is a catalytic subunit of two functionally distinct molecular complexes called mTORC1 and mTORC2. mTORC1 is composed of five proteins, mTOR, RAPTOR, mLST8, PRAS40, and FKBP38, and the complex performs its function by phosphorylating the p70S6 and 4E-BP1 kinases, thereby regulating the expression of proteins that promote cell proliferation and survival, such as c-Myc, cyclin D1, and STAT3. The mTORC2 complex includes RICTOR, MAPKAP1, PRR5/PRR5L, Mlst8, and Deptor. mTORC2 directly phosphorylates Akt and regulates the organization of actin cytoskeleton by phosphorylating PKC-α. Unlike mTORC1, which is sensitive to acute treatment with rapamycin (RAPA), mTORC2 is less sensitive to the drug, although chronic treatments were shown to disrupt the integrity and function of this complex (55). The mTOR-inhibitor *Everolimus* (EVR) was derived from *Sirolimus* (SRL) and both compounds bind FK506-binding protein 12 (FKBP12) in the cytoplasm. mTOR inhibitors (mTORi) were initially designed as anti-cancer drugs, as well as immunosuppressive agent, because of their ability to suppress the growth and proliferation of tumor cells in mice (56). Dysregulation of cell cycle characterizes several types of tumors, and consequently, mTOR became an important therapeutic target for cancer patients. mTOR inhibition leads to an arrest of cells in the G1 phase of the cell cycle and a severe reduction of protein synthesis (57), demonstrating that the mTOR pathway is crucial for cell survival and protein synthesis. Notably, hyperactivation of the mTOR/PI3K/AKT pathway is present in virtually all types of tumors as a main consequence of somatic loss of the PTEN phosphatase, which is mutated or epigenetically inactivated in an large number of cancers (58). Inhibition of angiogenesis and decreased VEGF synthesis (59) constitute additional relevant effects characterizing mTORi. In HPV-positive transplanted patients, lifelong immunosuppressive therapy with mTORi has been associated with a significant reduction in the incidence of *de novo* neoplasms (60). Of particular interest was the case of a young liver transplant patient in whom the conversion to SRL therapy was followed by a rapid regression of skin warts suggesting that mTORi may be beneficial in immunosuppressed patients with HPV-induced relapsing warts (61). In addition, post-transplantation treatment with Rapamycin was shown to reduce the ability of B cells to undergo EBV lytic cycle replication (62), a well-known factor predisposing to EBV-PTLD. In the same setting, it has been demonstrated that the combination of mTOR and inhibitors of HSP90 (a dysregulated protein in EBV-related PTLDs) had a synergistic effect in inducing apoptosis and *in vitro* cytotoxicity of EBV-positive cells (63), suggesting the possible therapeutic efficacy of this combination in the control of PTLD.

Table 1 summarizes the main effects exerted by the different classes of immunosuppressive drugs on immune cells.

**Table 1:** Main effects exerted by immunosuppressive drugs on immune cells.

**Mechanism of action**	**Drugs**	**Impact on immune cells**	**References**
Regulation of gene expression	Glucorticoides	• Impair monocyte and macrophage function • Decrease circulating levels of CD4+ T cells.	(22)
Inhibition of *de novo* purine synthesis	Azathioprine Mycophenolate Mofetil	• Interfere with T-cells stimulation and proliferation • Significant reduction of CD107 expression in NK cells • Significant reduction of INF-γ production by NK cells • Down-regulation of co-stimulatory and adhesion molecules human monocyte-derived DC	(22) (64) (65)
Kinase and phosphatase inhibitors	Calcineurin inhibitors mTOR inhibitors	• Inhibit of NF-kB phosphorylation in CD3^+^ T-cells, CD4^+^ T-cells and CD8^+^ T-cells • Prevent naïve T-cells differentiation • Preserve stable numbers of NK cells • Reduce IL-2 and TNF-α production by macrophages • Affect DC maturation *in vitro* • Impair IL-12 production by DCs • Impair B-cells proliferation • Impair CD19+CD27+ memory B-cells • Promote CD4^+^CD25^high^FOXP3^+^ Tregs expansion • Reduce the numbers of NK cells • Decrease M-MDSCs differentiation • Induce macrophages apoptosis • Impair DCs maturation and function	(66) (67) (68) (69) (70, 71) (72) (73) (74) (75) (68) (76) (77) (78–80)

## Involvement of Immune Cells in the Maintenance of Immune Competence and in the Control of Cancer Development in Life-Long Immunosuppressed SOT Patients

### B Cells

Despite currently used immunosuppressive regimens are mainly investigated for their effects on T lymphocytes, the impact of these treatments on B-cell function may also be of pathogenic relevance. In fact, in addition to their activity as the producers of antibodies, B cells also function as potent antigen presenting cells and therefore their impairment may negatively impact on the immune control of nascent tumors. MPA and rapamycin were shown to strongly inhibit the proliferation of purified human B lymphocytes stimulated by CD40 ± Toll-like receptor triggering, whereas TAC and CSA had only marginal effects (73). Moreover, MPA and rapamycin also inhibited immunoglobulin production, which was independent of the degree of B-cell stimulation, and induced apoptosis of B lymphocytes (73). These findings clearly indicate that MPA and Rapamycin are able to profoundly inhibit B cells responses. On the other hand, it has been shown that CNIs inhibit humoral immune responses by interfering with T helper signals and not by eliciting direct effects on B lymphocytes (81). Nevertheless, SRL and TAC have different effects on the proliferation, activation and differentiation of B lymphocytes. In particular, clinically relevant doses of SRL, but not of TAC, inhibited the CD19^+^CD27^+^ B cell memory compartment. Moreover, SRL effectively blocked B cell differentiation into plasma cells and decreased absolute B cell counts. Despite inhibition, the residual B cells that do respond to stimulation in the presence of SRL result in a population shift toward more activated phenotypes. These activated B cells are able to induce a robust allogeneic T-cell activation and proliferation and a shift toward a Th1 phenotype (74).

An emerging body of evidence indicates that B cells may have regulatory properties that contribute to induction and maintenance of tolerance. Regulatory B cells (Bregs) is a relatively newly recognized subset of B lymphocytes showing potent regulatory activities in different inflammatory and autoimmune settings and playing a critical role in preventing transplant rejection. The characteristic phenotype identifying Bregs is not yet clear, and this subset of B cells is mainly identified by IL-10 production (82). Although IL-10 release constitutes the main mechanism by which Bregs perform their immunosuppressive activity, these cells may also impair immune responses through other mechanisms and cytokines, including IL-35, Fas-Ligand, PD-L1, and TGF-β (83). These activities allow Bregs to inhibit both innate and adaptive immune response and promote the expansion of immunosuppressive regulatory T cells (Tregs). In the transplant setting, it has been shown that increased Breg cell frequency correlated with reduced rejection episodes and long-term allograft survival (83). Higher numbers of Bregs were detected in operatively tolerant patients (in the absence of immunosuppressive drugs) compared to pharmacologically immunosuppressed patients, suggesting a protective function of the Breg cell subset (84, 85). Many commonly used immunosuppressive drugs, such as CSA, TAC, Prednisolone, AZA, and MMF, were shown to reduce Breg numbers (83), having thus a negative impact on the ability of transplanted patients to control the tolerance to the allograft.

Globally, immunosuppressive drugs used in the transplant setting may affect B lymphocytes resulting in both anti-tumor and pro-tumorigenic activities (86), although it remains unclear whether the net effect has any contributory role to the increased risk of cancer in the transplant setting.

### T Cells

Immunosuppressive drugs are mainly known for their ability to inhibit the function and survival of T lymphocytes and the NFAT and NF-kB are the main signaling pathways targeted by these drugs. These effects are best exemplified by TAC, which, in addition to its ability to inhibit the calcineurin/NFAT pathway, was also shown to inhibit NF-kB activity and TNFα production in CD3^+^ T cells, CD4^+^ T cells and CD8^+^ cytotoxic T cells isolated from healthy donors (66). CNIs such as CsA and TAC may also prevent naive T cell differentiation into Th1, Th2, and Th17 subsets and inhibit the production of IFN-γ, IL-4, and IL-17 by memory CD4^+^ T cells (67). These effects could at least in part account for the increased risk of *de novo* cancer in SOT patients treated with CNIs (60). A recent study investigated the contribution of residual T-cell immune function in mediating the decreased incidence of SCC showed by renal transplant recipients switching CNI-based therapies to mTORi. While both RAPA and TAC enhanced the survival of OVA-expressing skin grafts in mice, and inhibited short-term antigen-specific CD8^+^ T cell responses, RAPA but not TAC induced a significant infiltration of CD8^+^ effector memory T cells into UV-induced SCC lesions. Moreover, only RAPA was able to increase the number and enhance the function of CD8^+^ effector and central memory T cells in a model of long-term contact hypersensitivity. In fact, RAPA was shown to promote the generation of long-lived memory precursors by altering the process of differentiation of short-lived precursors (87, 88). These findings are consistent with the possibility that the lower risk of *de novo* SCC showed by patients switched to mTORi regimens is probably due to enhanced CD8^+^ memory T-cell responses to new antigenic stimulations occurring in their skin (89).

**Figure 1:**
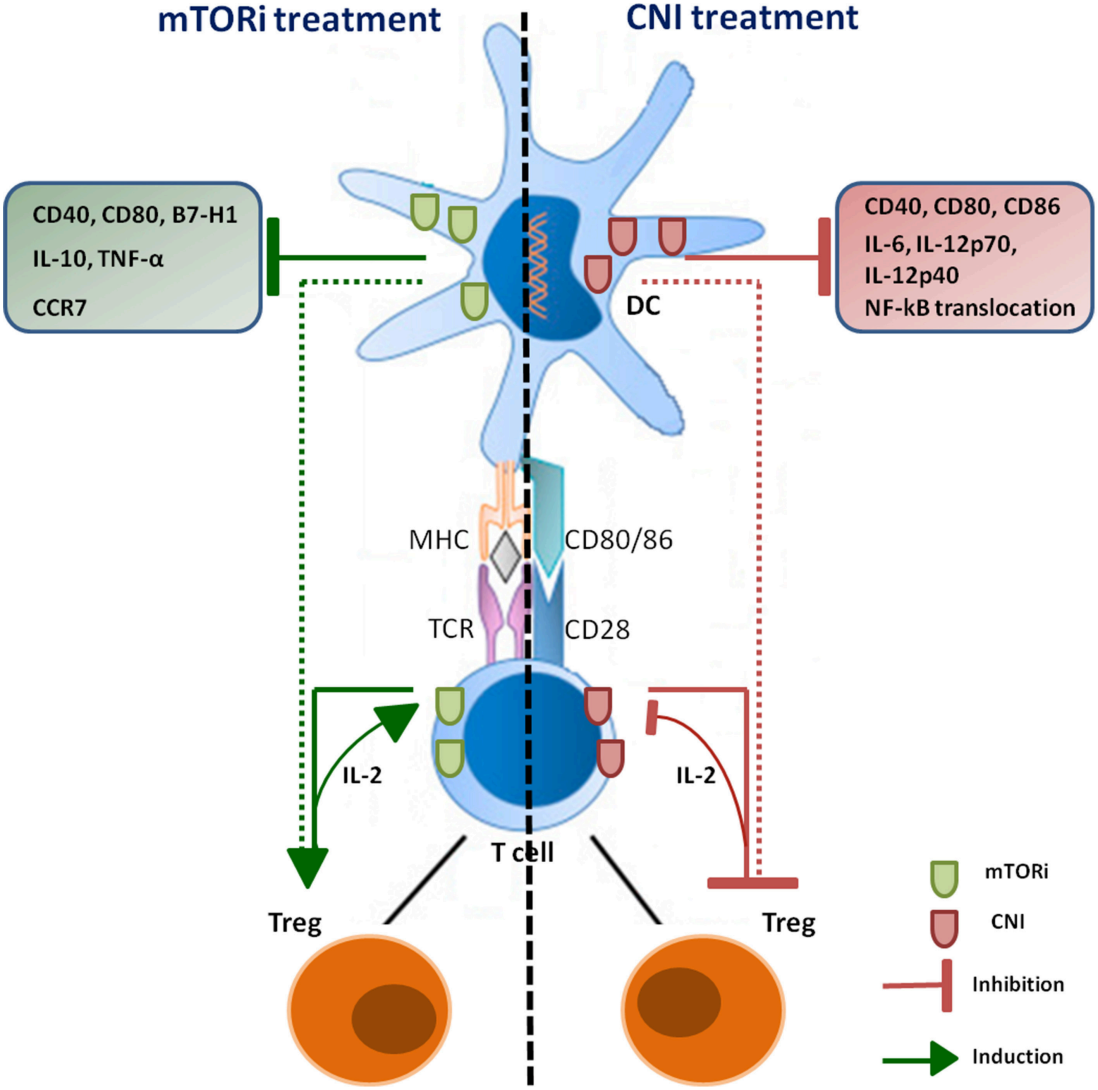
Schematic overview of CNI and mTORi immunosuppressive effects on DCs and Treg. Immunosuppressive agents have opposite effects on Treg population through both direct and indirect mechanisms. DCs treated with CNI show a down-regulation of IL-2 and IL-12 production, which are necessary to induce Treg proliferation; CNI also direct inhibit production and re-uptake of IL-2 by T lymphocytes, impairing differentiation and proliferation of Treg. RAPA-DCs promote Treg proliferation and are able to induce the generation of this subset; mTORi can also direct stimulate induction of Treg, promoting organ transplantation tolerance.

Due to the critical role of Treg cells in maintaining tolerance against self-antigens and controlling excessive immune responses, the effects exerted by immunosuppressive drugs on these cells were extensively investigated. Nevertheless, the possible promotion of cancer development associated with abnormal expansion or activation of Treg cells induced by these drugs remains an underexplored area. Treg cells are mainly characterized by the expression of CD25 and FoxP3 and represent 5–10% of all peripheral CD4^+^ T cells (90). Tregs can be divided into resting Tregs (CD45RA^+^FoxP3^low^), effector Tregs (CD45RA^−^FoxP3^high^) and cytokine-producing Tregs, (CD45RA^−^FoxP3^low^) (91). Tregs can be also classified into two sub-groups according to their development: natural Tregs, which develop during the normal process of T-cell maturation within the thymus and that are characterized by high expression of CD25, co-stimulatory molecule cytotoxic T-lymphocyte antigen 4 (CTLA4) and the tumor-necrosis factor (TNF)-superfamily member GITR (glucocorticoid-induced TNF receptor family-related protein (TNFRSF18); adaptive Tregs, which generate by populations of mature T cells under certain conditions of antigenic stimulation and show variable levels of CD25 expression depending on the disease setting and the site of regulatory activity (92). Treg cell mechanism of action involves immunosuppressive activities against other T cell subsets, B cells, macrophages, DCs and NK cells and the release in the microenvironment of immunosuppressive cytokines such as IL-10, IL-35, and TGF-β to prevent T-cell proliferation and maturation of antigen presenting cells (93). Treg cells may also secrete granzymes and perforins (94) and express CTLA-4, which may inhibit the activity of DCs (95). The pilot study by Levitsky et al. has shown that, in liver transplant patients, monotherapy with SRL resulted in a higher percentage of Tregs in peripheral blood compared to non-SRL monotherapy (96). These findings are consistent with the observation that the expression of FoxP3 requires IL-2, whose gene transcription is blocked by CNI but not by SRL. These data were confirmed in a subsequent study that showed that RAPA, but not CsA, promotes the induction of Tregs rather than inhibiting their function (97) (Figure 1). Of particular relevance in terms of potential increased risk of cancer development is the effect that RAPA has on Tregs. In fact, it has been demonstrated that immature dendritic cells (iDCs) treated with low-doses of RAPA and injected intravenously in rats are able to selectively expand CD4^+^CD25^+^Foxp3^+^ Tregs (98). RAPA was also shown to preferentially promote the expansion of CD4^+^CD25^high^FOXP3^+^ Tregs as compared to the CD4^+^CD25^neg^FOXP3^+^ Treg subset (75). Intriguingly, RAPA enhances the expression of CXCR4, the ligand of stromal-derived-factor-1 (SDF-1), which is constitutively expressed in the bone marrow, suggesting that this drug may promote the development of Tregs with distinct homing properties (99).

The reduced risk of skin cancer development observed in kidney transplant recipients treated with SRL as compared to those receiving CNIs was associated with different effects exerted by the two drugs on T cell populations infiltrating the skin. It has been shown that the treatment with SRL significantly increased the absolute number of CD4^+^ T cells, memory CD8^+^ and CD4^+^ T cells, and Treg cells in the sun-exposed skin compared to non-sun-exposed (100). Notably, no differences were found in the absolute number of any T cell subset were observed in the blood, suggesting that the percentage of T cell subsets detectable in the blood does not always accurately reflect the percentage of T-cell subsets in the skin of kidney transplant recipients.

Th17 cells are characterized by their ability to produce pro-inflammatory cytokines, including IL-17A, IL-17F, and IL-22, and are critical for host defense against pathogens but have also been implicated in causing autoimmune disorders and cancer, although their role in carcinogenesis is less well defined (101). Besides decreasing the proportion and function of CD4^+^ Tregs, CNIs up-regulate Th17 cell-associated pathways (102), which are involved in allograft rejection and may also contribute to the enhanced risk of *de novo* malignancies in SOT patients treated with these drugs. Available data indicate that the replacement of TAC therapy to SRL therapy suppresses Th17 activity and up-regulates the percentage of Treg cells in kidney transplant recipients. SRL inhibited Ser705 phosphorylation of STAT3 in CD4+ T cells, which promotes a differentiation switch toward Treg cells rather than to Th17 cells. The drug was also shown to induce a downregulation of IL-17 and an increased expression of Foxp3 in Th17 cells. Therefore, the Th17/Treg ratio modulation induced by conversion from TAC to SRL promotes a better control of the allograft (103) and may also contribute to the decreased risk of cancer associated with the use of SRL.

An increasing number of studies have focused the attention to a recently described subpopulation of T cells, the tissue resident memory T (Trm) cells, a subset of non-circulating lymphocytes that reside in multiple peripheral tissue sites, including lung, intestine, liver and skin. Trm cells are characterized by the expression of CD103, CCR7, CD28, and IL-7R and are CD45RA-CD69-. Although Trm cells were initially considered as early immune effectors in infectious diseases, recent studies highlighted their role in mediating therapeutically relevant immune responses against cancer (104). In the setting of SOT transplantation, available data point to an important role of Trm in the control of common chronic viral infections and site-specific acute infections (105), suggesting that these cells may contribute to some aspects of graft tolerance. Conversely, Trm cells can potentially mediate anti-allograft responses through their strong immunostimulatory abilities (106). The possible implications of Trm cells in the risk of cancer in SOT patients treated with immunosuppressive drugs remain to be elucidated. Nevertheless, the notion that CNI and mTOR inhibitors mainly target the early activation phases of T lymphocytes suggest that these drugs may have limited effect on Trm cells because of their pre-activated phenotype (107).

### NK Cells

NK cells are key components of innate immune system are able to mediate cell lysis without prior stimulation by antigens. Their activation is closely dependent on the balance between the expression of inhibitory molecules and triggering of activatory NK cell receptors by their cognate ligands. While in hematopoietic stem cell transplantation NK cells have been shown to play a significant role in the graft-vs.-leukemic effect, the role of NK cells in SOT is controversial due to conflicting clinical and pre-clinical data. In fact, NK cells were shown to worsen T-cell responses during allograft rejection, but also to promote tolerance induction under treatment with immunosuppressive drugs (108). In a murine kidney transplant model based on hybrid resistance, NK cells were shown to mediate long-term allograft injury even in the absence of T and B cells (109). Preclinical data indicate that immunosuppressive agents may affect the number and function of NK cells. In an elegant work, Aislin Meehan et al. have shown that treatment of PBMCs from healthy donors with different immunosuppressive drugs resulted in impairment of NK cell function that varied according to the type of drug investigated and the dose used. At concentrations used in the clinical setting, CsA and Prednisolone caused a significant reduction in NK cell expression of CD107 (a degranulation marker indicative of cytotoxic activity) and the production of IFN-γ (64). The activity of NK cells is also inhibited by treatment with RAPA, as shown by the group of Wai et al. who analyzed the proliferation and potential cytotoxicity of rat NK cell lines in the presence of different types of immunosuppressive drugs. NK cell number and function remained stable in graft recipients treated with CsA and FK506, whereas RAPA significantly inhibited proliferation and cytotoxicity of NK cells (68). On the contrary, Monteau et al. demonstrated that both CsA and TAC have a strong negative impact on degranulation and IFN-γ production of by NK cells *in vitro* (110). Overall, available data seem to indicate that the effect of immunosuppressive drugs on NK cells is not as strong as that induced on T lymphocytes, at least *in vivo* (108). Considering the emerging contributory role of NK cells in the control of primary tumors and particularly of cancer metastasis (111), it will be of relevance to monitor NK cell number and function in SOT patients at risk of *de novo* malignancy.

#### Myeloid-Derived Suppressor Cells

Myeloid-derived suppressor cells (MDSCs) are innate cells that play a pivotal role in inhibiting T-cell dependent responses. MDSCs are not a terminally differentiated cell population and are characterized by CD33^+^ expression, whereas CD3, CD14, CD19, CD56, and HLA-DR are usually negative. In mice, MDSCs consist of two large groups of cells termed granulocytic or polymorphonuclear (PMN-MDSCs) characterized by CD11b^+^Ly6G^+^Ly6C^lo^ expression, and monocytic (M-MDSCs) characterized by CD11b^+^Ly6G^−^Ly6C^hi^ expression (112). Also in human, two main groups of MDSCs have been identified: granulocyte MDSCs (G-MDSCs, CD33^+^CD11b^+^CD14^−^CD15^+^) and monocytic MDSCs (M-MDSCs, CD33^+^CD11b^+^CD14^+^CD15^−/low^) (112, 113). M-MDCSs contributes to production of inflammatory cytokines and growth factors, which may have a strong immunosuppressive effect, including the inhibition of T-cell proliferation (112, 114), and impaired maturation and development of DCs. In fact, DCs generated in the presence of MDSCs were found to be less effective in antigen uptake, migration and induction of IFN-γ production by T cells (115). Furthermore, MDSCs are able to down-regulate the production of IL-12 by macrophages while increasing IL-10 production in response to cell-cell contact (116). Experiments carried out in mouse models demonstrated that MDSCs identified with phenotypic biomarkers were able to inhibit proliferation, but not activation, of effector T cells and to induce apoptosis in a contact-dependent manner. Interestingly, CD4^+^CD25^high^FoxP3^+^ Treg cells were insensitive *in vitro* to MDSC-mediated suppression. These results suggest that increasing numbers of MDSCs can inhibit alloreactive T-cell proliferation *in vivo* and that these cells may participate in the maintenance phase of tolerance (114). In an analysis of a cohort of 50 kidney transplant patients, Meng et al confirmed that the number of MDSCs was associated with long-term graft survival and showed that this population regulates the imbalance between Tregs and Th17 cells (117). Moreover, in an experimental mouse model of transplantation, Garcia et al. showed that MDSC were associated with an increased accumulation of Foxp3-expressing Tregs in the allografts of transplant recipient mice following tolerogenic treatment (118). The impact of CsA on the myeloid population was investigated in a skin transplantation study showing that CsA can stimulate the accumulation of CD11b^+^Gr1^+^ cells (MDSCs in mice) by improving the immune responses through the NFAT pathway, also promoting the differentiation into CD4^+^ and CD8^+^ T cells. In a murine cardiac transplantation model, RAPA treatment led the recruitment of MDSCs, which also expanded as a consequence of the effects of the treatment on the mTOR pathway (119). In addition, inhibition of the mTOR signaling was shown to promote a shift of MDSCs toward the G-MDSCs subtype (120). A recent study in human kidney transplantation demonstrated that CD33^+^CD11b^+^HLA-DR^−^ MDSCs were able to expand Tregs *in vitro*, through the release of TGF-β and IL-10 (121). RAPA was also shown to decrease M-MDSCs differentiation from myeloid progenitors by blocking the glycolytic metabolic pathway (76). An excessive expansion of MDSCs promoted by immunosuppressive drugs may contribute to increase the risk of cancer in SOT patients. In fact, MDCSs constitute a critical component of the immune suppressive niche characterizing tumor microenvironment, where these cells may promote immune escape and malignant progression by affecting both the innate and adaptive immune responses (122). Studies carried out so far in the SOT setting focused mainly on the role of MDSCs in the induction of tolerance and allograft control and no data are currently available with regard to the possible correlation between expansion/overactivity of MDSCs and risk of the novo malignancy in these patients. Some indirect clues derived from a study carried out in a mouse neuroblastoma-bearing chimeras, which showed that adoptive recipient leukocyte infusion enhanced anti-tumor responses of allogeneic bone marrow transplantation. These anti-tumor effects, however, were counteracted by expansion of host MDSCs pointing to a relevant role of these immunosuppressive cells in limiting the efficacy of anti-tumor immunity in the transplant setting (123).

#### Macrophages

Macrophages were identified by Elie Metchnikoff as an essential component of innate immune system that forms the first line of defense against pathogens (124). According to their differentiation status and functional role in the immune system, macrophages are conventionally classified into M1 and M2 subtypes, although these cells has the ability to differentiate into a variety of phenotypes in response to different stimuli from the microenvironment (125). The M1 phenotype can be identified by the overexpression of surface molecules such as MHC-II and CD86, and increased ability to present antigens and kill intracellular pathogens. *In vitro*, classical activation can be induced by stimulating macrophages with IFN-γ and LPS, causing TNFα production, associated with microbicide activity, production of pro-inflammatory cytokines and cellular immunity. Macrophages then undergo a process of activation toward a pro-inflammatory phenotype, increasing their ability to kill intracellular pathogens and contributing to the progression of the inflammatory process. M2 macrophages are generated in the presence of the anti-inflammatory cytokines IL-4, IL-10, and IL-13 and participate in tissue remodeling and long term repair (126). In addition to the host defense role, macrophages play an important role in homeostasis, and pathological processes such as obesity and malignancy (127). In particular, they show a more immunosuppressive phenotype characterized by impaired antigen presentation to T cells and production of cytokines that stimulate a Th2 response (128). Evidence accumulated so far however indicates that the binary classification of macrophages in M1 and M2 subtypes is an oversimplification that does not account for the marked plasticity of these cells that may acquire a broad and continuous spectrum of different phenotypes, with M1 and M2 representing the two extremes (128). Examples of non-M1/M2 cells are: CD169^+^ macrophages, detected in bone marrow, lymph node, liver, and spleen, and mainly involved in erythropoiesis and immune regulation; macrophages expressing TCRαβ or TCRγδ identified in inflammatory and infectious diseases; a novel subtype of tumor-associated macrophages characterized by an M2-like immunosuppressive gene profile and expressing a novel receptor “macrophage receptor with collagenous structure” (MARCO), detected in mouse tumor models of mammary carcinoma, colon cancer and B16 melanoma (128). The function of these non-M1/M2 macrophages however remain far from being fully characterized. In the SOT setting, macrophages play a controversial role: the presence of CD86^+^ macrophages is associated with acute rejection in kidney transplants (129) and in mouse models of heart transplant (130). Likewise, macrophages may exert anti-inflammatory responses and immunosuppressive effects that help maintain the peripheral tolerance, being able to produce IL-10 and TGF-β, involved in the resolution of graft inflammation (131). The use of immunosuppressive drugs has also an impact on the macrophage compartment: the use of CsA and TAC was associated with a decrease in the production of inflammatory mediators, such as IL-2 and TNF-α (69). Moreover, CNIs were shown to promote the differentiation of macrophages to the M2 phenotype (132). CsA was shown to impair the phagocytosis activity of macrophages and enhance the severity of infectious diseases (133). mTORi were shown to significantly decrease the production of chemokines induced by LPS, such as MCP-1, IL-8, RANTES, MIP-1α, and MIP-1β and their combination with glucocorticoids increased the production of the anti-inflammatory cytokine IL-10 through the STAT3 transcription factor (134). mTORi could also induce macrophage apoptosis, mainly affecting the M2 rather than M1 subset (77). It has also been documented that the administration of MMF reduces the synthesis of nucleotides by inhibiting the inosine monophosphate dehydrogenase pathway (135), decreases the production of IL-1β, IL-10, TNF-α (134) and the expression of adhesion molecules (136) by macrophages. Studies aiming at assessing the role of macrophages in SOT patients with cancer are scarce. No significant difference in the density of tumor-associated macrophages was observed in SCCs from SOT recipients as compared to SCC of non-transplant patients. These findings seem to rule out that the density of these cells may contribute to a worse prognosis of invasive SCC in transplant patients. Intriguingly, however, the density of tumor-associated macrophages infiltrating SCC *in situ* was markedly lower than that detected in non-transplant patients (137). It remains to be elucidated whether this is the result of direct effects of immunosuppressive drugs and whether this decreased infiltration of macrophage contributes to the enhanced invasiveness of SCC in SOT patients.

#### Dendritic Cells

DCs constitute a heterogeneous population of APCs that mediates critical connections between innate and adaptive immunity (138). These cells play a pivotal role in antigen processing and presentation resulting in the induction of effective immune responses against pathogens and tumor cells. Notably, these cells are the most powerful APCs, being able to orchestrate a primary immune response but also to induce immune tolerance (138). In particular, DCs play a central role in priming naive T and B cells, the first critical steps in the induction of an antigen-specific immune response. DCs develop from CD34^+^ bone-marrow progenitor cells or from CD14^+^ monocytes and differentiate into immature DCs (iDCs), which are functionally specialized to take up exogenous antigens. After recognition, exogenous antigens are internalized and the activated DCs migrate to the draining lymph node, where they can induce an adaptive immune response (139). This successful outcome requires the processing of antigens and their loading in the form of small peptides on the main histocompatibility complex (MHC) molecules. Peptides loaded on MHC-II molecules are recognized by antigen-specific CD4^+^ T helper cells, while peptides loaded on MHC-I molecules are recognized by antigen-specific CD8^+^ T lymphocytes. The presentation of internalized antigens on MHC-I molecules is defined as cross-presentation, a crucial process in the induction of effective adaptive immune responses against tumors and viruses that do not infect DC directly and that may induce peripheral tolerance (140). The MHC-I pathway is normally used to present endogenous antigens and cross-presentation is particularly important because it allows DCs to present through the MHC-I pathway also exogenous antigens, which are usually mainly presented by MHC-II molecules (141). Antigen cross-presentation involves two main pathways: (1) the vacuolar pathway, in which the processing/loading takes place within the endo/lysosomal compartment and (2) the endosome pathway, in which the internalized antigens are transported from the endosome to the cytosol where they are degraded by the proteasome (142). The co-stimulatory CD40/CD40L axis along with the danger signal provided by an exogenous antigen are catalysts for DC licensing. Therefore, exogenous antigen cross presentation and the consequent activation of naive CD8^+^ cytotoxic T cells, provide the immune system with an important mechanism for generating immunity to viruses while preserving tolerance to self (143).

DCs exist in two differentiation states, each of which has distinct phenotypic, morphological and functional characteristics: immature dendritic cells (iDCs) and mature dendritic cells (mDCs). iDCs phenotypically show high expression of CCR1, CCR5, CCR6, and CD68, while the expression of CCR7, CD86, CD80, CD40, and CD83 is low. These cells are able to capture and process the antigens they encounter and subsequently evolve to mDCs, which are characterized by high expression levels of CD83, CD86, CD49, CD80, CCR7, MHC-II, CD1a, and CD11c (144).

Another classification of DCs is based on different DC subpopulations: classical DCs (cDCs), plasmacytoid DCs (pDCs), monocyte-derived DCs (moDCs), and Langherans cells (LCs). cDCs are professional APCs that play a key role in shaping appropriate adaptive immune responses (145). There are two major subsets of cDC: cDC1, which are HLADR^+^CD11c^+^CD9α^+^ and CD24^+^ or CD103^+^ and require the transcription factors IRF8 (146), Batf3 (147), Nfil3 (148), and Bcl6 (149) for their development. These cells also express the CD141^+^ or blood DC antigen 3 (BDCA3). This population was found in spleen, tonsil, liver, lung and skin. Similar to mouse DCs, human cDC1 CD141^+^ express TLR3, but unlike the mouse subset, they lack the TLR8 and TLR9. This population is particularly efficient at cross-presentation of cellular antigens and are highly competent to stimulating allogenic or autologous CD4^+^ T cell immune responses. The second major cDC subset, cDC2, expresses HLADR^+^ CD11c^hi^ and the IRF4 transcription factor. This subset is present in lymphoid tissues and CD141 molecule is expressed by splenic cCD2. Human blood cDC2 express high levels TLR2 and TLR8 and very low levels of TLR4. They are able, without TRL activation, to cross-present soluble antigens, a process that may be enhanced bafilomycin-mediated inhibition of endosomal acidification (145, 150). From an ontogenetic point of view, available data are consistent with the existence of common DC progenitors that give rise to pDCs and intermediate precursors of cDCs (pre-cDCs) that may differentiate into CD1c^+^ (BDCA-1) or CD141^+^ (BDCA-3) cDCs. In human peripheral blood mononuclear cells, the HLA-DR^+^CD14^−^CD11b^−^ subpopulation includes the CD1c^+^ DC subset (CD172α^+^ and IRF4^+^), the CD141^high^ DC subset (Clec9A^+^, XCR1^+^, IRF8^+^, and TLR3^+^), and the pDC subset (BDCA-2^+^, BDCA-4^+^, and CD123^bright^).

pDCs correspond to a subset of immature CD11c^−^ population distinct from classical myeloid CD11c^+^ DCs. Pre-pDCs express CD4 but lack T-cell receptor alpha (TCRα), TCRβ, TCRγ, TCRδ, or CD3 chains and are negative for B-cell lineage (CD19, CD21) or myeloid (CD13, CD14, CD33) markers. This population is characterized by the production of type I interferon during viral infection as they express constitutively IRF7. pDCs circulate in the blood and in peripheral organs and are strongly dependent on Fltl3 ligand (Flt3L), a potent endogenous DC growth factor, for their development and function. After maturation in response to TLR-ligation or CD40-engagement, pDCs became capable to present antigens to T cells and preferentially, induce generation of a unique type of CD8^+^ Treg cells (151).

LCs are the DCs of the epidermidis, although they may be also present in other stratified epithelia, such as the mucosal oral and vaginal epithelium. LCs share with cDCs common myeloid or monocytic progenitors. LCs are stellate cells expressing MHC-II molecules and the integrin αX chain CD11c. They express also CD1a and CD1c, two MHC-I-related molecules involved in the presentation of lipid antigens. This population is considered sentinel tissue-resident DCs and their development is independent of the Flt3/Flt3L axis stimulation (152).

moDCs are phenotypically difficult to differentiate from cDCs because they share the expression of CD11c, CD11b, and MHC-II, but they express the Fc-gamma receptor 1 (FcγRI) (153). These cells also express CD1α, which is characteristic of the DCs derived from monocytes. Their mature phenotype is characterized by the loss of CD14, a marker of differentiated monocytes and by an increase in the expression of CD1α, MHC-II, CD83, and CD86. moDCs can be successfully grown in the presence of GMC-SF and IL-4 and are capable of induce effective antigen-specific immune responses (154).

Immunosuppressive drugs employed in the management of allograft rejection in SOT patients can influence the phenotypic and functional characteristics of DCs, suggesting that their impaired function may also play a role in the development of tumors or in promoting the reactivation of oncogenic viral infections in immunosuppressed patients. MMF was shown to down-regulate co-stimulatory and adhesion molecules, such as CD40, CD54, CD80, and CD86, in human monocyte-derived DC *in vitro*, a decreased production of TNF-α, IL-10, IL-12, and IL-18, as well as less efficient stimulation of alloreactive T cells (65). Similarly, the 1α,25-Dihydroxyvitamin D3 (1,25(OH)2D3), i.e., the active form of vitamin D3, inhibits the differentiation and maturation of human DCs, leading to down-regulated expression of CD40, CD80, and CD86 costimulatory molecules and inhibition of alloreactive T cell activation (155). Interestingly, Gregori et al. showed that short-term treatment of transplant recipient mice with 1,25(OH)2D3 in combination with MMF induced tolerance to islet allografts and expanded CD4^+^CD25^+^ Treg cells that were shown to adoptively transfer transplantation tolerance. Moreover, these DCs displayed a tolerogenic phenotype characterized by down-regulation of CD40, CD80, and CD86 and reduced IL-12 production (156).

The effects of CNIs on DCs are well-recognized and several groups have investigated the impact of these drugs on mouse and human DCs. TAC and CsA were found to inhibit the allostimulatory capacity of *in vitro*-generated myeloid DCs without affecting DC maturation (70). On the contrary, the pioneering work of Lee et al has shown how the treatment with CsA impaired the allostimulatory capacity of *in vitro* generated mouse bone marrow-derived DCs by downregulating CD40, CD80, and CD86 expression associated with reduced nuclear translocation of NF-κB, a transcription factor promoting DC maturation (157, 158). In addition, CsA inhibited DC-dependent production of INF-γ, IL-2, and IL- 4 by T cells, and IL-6, IL-12p40, and IL-12p70 by DCs (72). Recently, it was demonstrated that DCs generated in the presence of CsA lose their ability to induce Treg proliferation, with a strong reduction of IL-12 secretion and particularly of IL-2, which is necessary for the proliferation of CD4^+^CD25^+^Foxp3^+^ T cells (159), thus having a negative impact on this population (160). Moreover, TAC was shown to inhibit the ability of DCs to stimulate T cells and to decrease the production CXCL10 and IL-12. Although IL-12 production by DC was impaired by TAC, these cells did not promote Th2 development as T cells stimulated by TAC-treated DCs produced less interferon IFN-γ, IL-4, and IL-10 (161) (Figure 1).

In mouse models, RAPA strongly impacts DCs maturation and function resulting in the induction of well-defined phenotypic characteristics of these cells. Data accumulated so far indicate that RAPA is able to impair maturation of DCs, reduce DCs costimulatory molecule upregulation, decrease the production of pro-inflammatory cytokines, inhibit T cells stimulatory capacity (78) and promote the selectively development of Treg cells in animal models of solid organ transplantation (162) (Figure 1). Mouse and human DCs treated with RAPA have an immature phenotype with low levels of CD80 and CD40 receptors and with a decreased expression of B7-H1, the PD-L ligand, a negative regulator of T cells activation and inducer of peripheral tolerance (163).

During the maturation process, DCs downregulate the CCR1 and CCR5 receptors and upregulate the expression of CCR7, which promotes the DCs migration from the peripheral tissues to the sites where they will encounter the naive T cells for their priming (144). In both mouse and human DCs, immunosuppressive drugs have divergent effects on the modulation of chemokine receptors (CKR) in maturing DC, important in regulating DCs localization and homing (164). In an elegant study, Sordi et al. showed how the use of immunosuppressive drugs may interfere with the generation of effective immune responses by affecting DC function. In particular, these authors investigated the functional relevance of CKR in the process of DC maturation both *in vitro* and *in vivo*. CsA and TAC were shown to slightly modulate the expression of CCR7 but without affecting the function of this CKR. In contrast, RAPA increased the expression of CCR7 at the mRNA and protein level and enhanced the *in vitro* migration of human DCs to CCL19 and of mouse DCs to lymph nodes *in vivo*, suggesting that it could be due to the inhibition of endogenous IL-10 production (165), which has an inhibitory activity on DCs maturation (166).

RAPA-treated alloantigen-pulsed DCs were shown to induce antigen-specific regulation and prolong experimental heart allograft survival. Taner et al. demonstrated that RAPA-exposed DC loaded with donor cell lysates and their adoptive infusion prior to experimental heart transplantation prolonged fully MHC mismatched murine heart allograft survival (167). Notably, RAPA-treated DCs were also shown to stimulate the generation of antigen-specific Foxp3^+^ Treg cells thereby promoting organ transplant tolerance (168). The induction of tolerance by RAPA is further enhanced by the combination with Flt3L, which promotes the generation of tolerogenic, immunosuppressive DCs along with the production of CD25^+^Foxp3^+^ Treg cells and IL-10 secretion (169).

T cell responses are modulated by cytokines secreted by mDCs including pro-inflammatory cytokines, such as IL-12 and INF-α, and anti-inflammatory cytokines, such as IL-10. DCs treated with RAPA have a distinct cytokine secretion profile at different stages of maturation. In iDCs, RAPA reduced IL-10 and IL-12 production after LPS stimulation and increased apoptosis, while mDCs are resistant to RAPA-induced apoptosis, but they also show decreased production of IL-10 and TNF-α (79). A recent study demonstrated that relevant concentrations of RAPA (20 ng/ml) inhibit the ability of both TLR7- and TLR9-activated pDCs to stimulate the production of IFN-γ and IL-10 by allogeneic T cells. On the contrary, RAPA-treated TLR7-activated pDCs were capable of stimulating the activation of naive and memory T cells, while also stimulating the generation and proliferation of CD4^+^ FoxP3^+^ Treg cells (80).

In recent years, tolerogenic DCs (t-DCs) attracted a growing interest for the development of new strategies to prevent and control allograft rejection after SOT. Enhanced generation of t-DCs, however, may negatively impact the induction of antitumor immune responses in this setting. There are two main critical factors that could shape DC functions into immunologic or tolerogenic: the stage of differentiation and the environmental factors that DCs encounter. Indeed, t-DCs comprise most immature DCs and DCs with different maturation states. One of the major hallmarks of t-DCs is their ability to induce tolerogenic responses to a much greater extent and in a shorter time as compared with immature DCs. t-DCs exert a unique immune surveillance function and normally express low levels of MHC and costimulatory molecules (CD80, CD86, CD40, OX40L) on their surface, and display a low capacity of activating T cells, which is potentially associated with T cell anergy and increased Treg cell generation. Moreover, t-DC can express high levels of inhibitory molecules such as programmed death ligand (PD-L)-1 and PD-L2, leukocyte Ig-like transcripts (ILTs) 2,34, HLA-G and galectins that may contribute to their tolerogenic potential (170–172).

Another subpopulation of t-DC was shown to have a semi-mature phenotype characterized by the high expression of MHC class II and B7 costimulatory molecules and a low production of proinflammatory cytokines, such as IL-1β, IL-6, IL-12p40, and IL-12p70 or TNF-α. However, mature DCs can also became tolerogenic. Indeed, in a subset of both mature and immature monocyte-derived DCs, Munn DH et al., demonstrated the expression of indoleamine 2,3-dioxygenase (IDO), a negative regulator of T-cell proliferation. The authors hypothesized that the presence of IL-10 during DC maturation (a regulatory cytokine associated with the development of tolerogenic DCs) prevented IFN-γ-induced down-regulation of IDO, resulting in sustained expression of functional IDO even in mature, IFN-γ-activated DCs (173). IDO and t-DC induction were also found to be associated with spontaneous renal allograft acceptance (174).

Gregori et al identified a novel subset of t-DC, named DC-10, which secretes high levels of IL-10, express ILT4 and HLA-G, and have the specific function to induce Type 1 T regulatory (Tr1) cells which are critical in maintaining tolerance to self- and non-self-antigens (172). There is also a unique subset of human DC, characterized by high expression of CD11b and low expression of MHC class II, which can negatively regulate immune responses. These DC have high expression of Fas that can inhibit CD4^+^ T-cell proliferation and produce IL-10 and IP-10 via ERK-mediated inactivation of GSK-3 and subsequent up-regulation of β-catenin (175). pDC were also found to exert tolerogenic functions. In particular, these cells display heterogeneous properties with regard to antigen presentation, maturation and expression of different costimulatory molecules. Indeed, pDCs do not induce strong T-cell responses, but they were found to induce IL-10-producing regulatory Tr1 cells *in vitro* (176). Moreover, Abe M et al. reported the ability of pre-pDC to prolong vascularized organ graft survival promoting tolerance (177). A subset of pDCs expressing CCR9 was identified by Hadeiba et al. in resting secondary lymphoid tissues. This population was a potent enhancer of Treg cell function and effectively prevented acute graft-vs.-host disease induced by allogeneic CD4+ donor T cells. In support of the role of pDC in tolerance induction, some other studies suggested a possible functional correlation between the enhanced presence of pDC and the elevated frequency of Treg cells (178) with successful withdrawal of immunosuppression in liver transplant tolerance (179).

Immunosuppressive factors and molecules expressed by T cells can also shape DC into a more tolerogenic state. Indeed, IL-10-producing Treg cells suppressed DC maturation and prevented Th1 cell differentiation. Moreover, interaction between t-DCs and Tregs is essential for the establishment of immune tolerance induced by apoptotic cell administration. Indeed, Wu et al. demonstrated that t-DCs promote the expansion of Tregs via PD-L1 on their surface, and Tregs facilitate t-DCs to sustain tolerogenic state via IL-10 and TGF-β (180). Another example of DC-tolerance induction by T cells occurs via Foxp3^+^ Tregs that inhibit the expression of MHC-II molecules in DC that were not able to make cognate interactions with CD4+ T cells (181). In addition, regulatory DCs can selectively recruit Th1 cells and inhibit Th1 proliferation, and promote the generation of IL-4-producing alternative memory CD4 T cells with suppressive activity (182).

The possible direct role of immunosuppressive drugs on DC function in SOT patients with or without cancer is difficult to assess. The extent and quality of T-cell responses specific for epitopes of tumor-associated antigens or oncogenic viruses may constitute a surrogate marker of the function of DC in various transplant settings. This possibility is best exemplified by the clinical relevance of the monitoring of EBV-specific T-cell responses in transplanted patients at risk to develop EBV-associated PTLD (183).

## Concluding Remarks and Future Perspectives

In the last decade, the number of patients undergoing solid organ transplantation and those living and aging with a functional transplant have been progressively increased. This situation is posing new challenges in terms of optimization of immunosuppressive regimens to prevent allograft rejection while reducing the incidence of *de novo* malignancies. This issue is of increasing clinical relevance considering that the occurrence of a *de novo* malignancy in this setting is associated with a poor prognosis. In fact, tumors in transplanted patients may be more frequently diagnosed at advanced stages, suggesting a more aggressive behavior of the disease in immunocompromised patients (184). Moreover, several studies reported decreased overall survival in transplanted patients (185–187). A recent study demonstrated that the all-cancer 10-year survival in a large cohort of Italian liver transplant patients experiencing a malignancy was significantly lower as compared to that of liver transplant recipients without cancer (43 vs. 70%, HR = 4.66). The difference in survival was observed for both early and late mortality, although the effect was more pronounced in the first year after cancer diagnosis (188). These findings clearly point to the need to perform close oncologic monitoring during the post-transplant follow-up in order to ensure early cancer diagnosis and to improve survival. Indeed, a study carried out in more than 7,000 SOT patients indicated that rigorous cancer screening programs were effective in diagnosing at early stages lung, breast and prostate cancers (189). In addition to the conventional clinical follow up approaches for an early diagnosis of cancer, strategies able to identify transplanted patients at increased risk to develop a *de novo* malignancy are needed. Quantification of EBV DNA load in the blood, coupled with assessment of EBV-specific T-cell responses proved to be useful to identify transplanted patients at risk of impending EBV-associated PTLD (190). Nevertheless, for the large majority cancers arising after SOT, no suitable biomarkers are available to estimate the risk of *de novo* malignancy in this setting. A better understanding of the various mechanisms through which the different immunosuppressive regimens impair cancer immunosurveillance is required to develop suitable and effective immunomonitoring strategies for SOT patients. As described above, the immunosuppressants used to prevent allograft rejection have a heterogeneous and often complex impact on the function of immune cells. Dissection of this heterogeneity will be useful to understand why some drugs are associated with a decreased risk of a *de novo* malignancy as compared to others, as shown for mTORi (191, 192). Notably, these drugs used at immunosuppressive doses were also shown to efficiently synergize with an optimal oncological treatment to improve survival of patients with *de novo* carcinoma (193).

In perspective, a more thorough characterization of the effects induced by immunosuppressive drugs on the various DC subsets may give insights on the mechanisms underlying the different ability of these drugs to affect DC-dependent anti-tumor immune response and tolerance. Advanced monitoring of the effects induced by immunosuppressive regimens on the number and function of other immune cells, particularly Tregs and MDSCs, may be also relevant in this setting. This can be achieved by exploiting the power of new technologies such as mass cytometry, which allows for a high-dimensional single-cell analysis of the immune system through the simultaneous measurement of over 40 markers on individual cells (194). This technology has been recently proposed for the assessment of immune reconstitution after hematopoietic stem cell transplantation (195) and may be also useful if applied to SOT patients monitoring to simultaneously assess the phenotypic and functional features of multiple immune cell populations. Considering that the repertoire of B- and T-lymphocyte antigen receptors undergo significant changes during cancer development, the exploitation of novel methods for analyzing or evaluating these immune repertoires may facilitate the development of diagnostic and monitoring tools also in the SOT setting. Indeed, the high-throughput sequencing of immune repertoire technology, which provides a robust tool for deep sequencing repertoires of B- and T-lymphocyte antigen receptors, has been applied to the identification of tumor biomarkers and the development of immunotherapeutics for cancers (196). Integrating these novel technologic approaches with existing immune monitoring techniques will allow a better understanding of immune regulation in SOT recipients and lead to new opportunities to improve patient outcome.

## Author Contributions

MC and RD designed and wrote the manuscript. All the authors revised and approved the manuscript.

### Conflict of Interest Statement

The authors declare that the research was conducted in the absence of any commercial or financial relationships that could be construed as a potential conflict of interest.
